# Supplementary material for: Acoustic Enrichment of Heterogeneous Circulating Tumor Cells and Clusters from Metastatic Prostate Cancer Patients
Source: Anal Chem. 2024 Apr 24;96(18):6914–21. doi: 10.1021/acs.analchem.3c05371 (PMC11079855; doi:10.1021/acs.analchem.3c05371)
Supplement: Supplementary file 1 — ac3c05371_si_001.pdf [file ac3c05371_si_001.pdf]

Supporting Information

Acoustic enrichment of heterogenous circulating tumor cells and clusters from metastatic prostate cancer patients

*Cecilia Magnusson [a], Per Augustsson [b], Eva Undvall Anand [b], Andreas Lenshof [b], Andreas Josefsson [c,d,e], Karin Welén [c], Anders Bjartell [f], Yvonne Ceder [g], Hans Lilja [a, h], and Thomas Laurell [b]

[a] Department of Translational Medicine, Lund University, Lund SE-22100, Sweden.

[b] Department of Biomedical Engineering, Lund University, Lund SE-22100, Sweden.

[c] Institute of Clinical Sciences, Department of Urology, Gothenburg University, Gothenburg SE-41345, Sweden.

[d] Wallenberg Center for Molecular Medicine, Umeå University, Umeå SE-90187, Sweden.

[e] Department of Urology and Andrology, Institute of surgery and perioperative Sciences, Umeå University, Umeå SE-90185, Sweden.

[f] Department of Translational Cancer Research, Lund University, Lund SE-22100, Sweden.

[g] Department of Laboratory Medicine, Lund University, Lund, SE-22100 Sweden.

[h] Department of Pathology and Laboratory Medicine, Surgery (Urology), and Medicine (GU Oncology), Memorial Sloan-Kettering Cancer Center, New York, NY 10065, U.S.A.

*Corresponding Author: Cecilia.Magnusson@med.lu.se

Table of Content

Table S1, with patient information

Figure S1: describing the acoustophoretic principle.

Figure S2: additional cell separation performances in the acoustic chip

Figure S3: antibody control staining

Supplemental note describing Acoustic cell separation theory and cell movement as well as data analysis.

Table S1. Prostate cancer patient information overview

**Patient #**	1	2	3	4	5	6	7	8	9	10	11	12
**Hospital site**	Gothenburg	Malmö	Malmö	Malmö	Malmö	Malmö	Gothenburg	Gothenburg	Malmö	Malmö	Malmö	Malmö
**Date of diagnosis**	okt-19	aug-21	jan-22	apr-22	juni-22	sep-22	mars-18	jan-20	okt-22	okt-22	aug-21	dec-21
**Date of sample analysis**	sep-20	nov-21	mars-22	maj-22	sep-22	nov-22	nov-22	nov-22	nov-22	nov-22	nov-21	feb-22
**Age range at analysis (Years)**	90-94	75-79	70-74	75-80	65-69	55-59	75-79	75-79	90-94	80-84	80-84	64
**TNM**	T4NxM1	T3T4N1M1	T4N1M1	T2N1M1	T4N1M1	T3N1M1	TxNxM1	T3NxM1	TXN1M1	T3N1M1	T3N1M1	TXN1M1
**Gleason score**	4+5	4+5	4+5	N.A.	4+5	4+5	4+4	N.A.	N.A.	4+5	4+5	4+5
**Location of metastasis**	bone	LN, bone	LN, bone	LN, bone	LN, bone	LN, bone, lung	bone	bone	LN, bone	LN, bone	LN, bone	LN, bone
**PSA level sample analysis (ng/mL)**	770	0.81	397	476	112	20	490	150	1015	9.0	20	3.4
**PSA level diagnosis (ng/mL)**	1100	60	3518	6647	118	312	170	1700	1015	989	26*	167
**Alkaline phosphatase level**	19.3	2.7	38	8.7	1.5	2.0	1.6	11	3.4	12	1.9	1.7
**Treatment**	GnRH**	GnRH**	GnRH**	GnRH**	GnRH**<br>Bikalutamid	GnRH**	GnRH**<br>***	GnRH**<br>****	GnRH**	GnRH**<br>Bikalutamid<br>Abiraterone	GnRH**<br>Bikalutamid	GnRH**<br>Bikalutamid
**CellSearch**	Y	Y	Y	Y	Y	Y	Y	Y	Y	Y	N	N

* PSA level 2 months prior to diagnosis

** Any GnRH-analogue, in some patients preceded by one- or two- months GnRH-antagonist

*** Patient 7 recieved also other treatments, but not at the time of sample analysis (Docetaxel, Enzalutamide, Radium).

**** Patient 8 recieved also other treatments, but not at the time of sample analysis (Abiraterone).

LN = lymph node

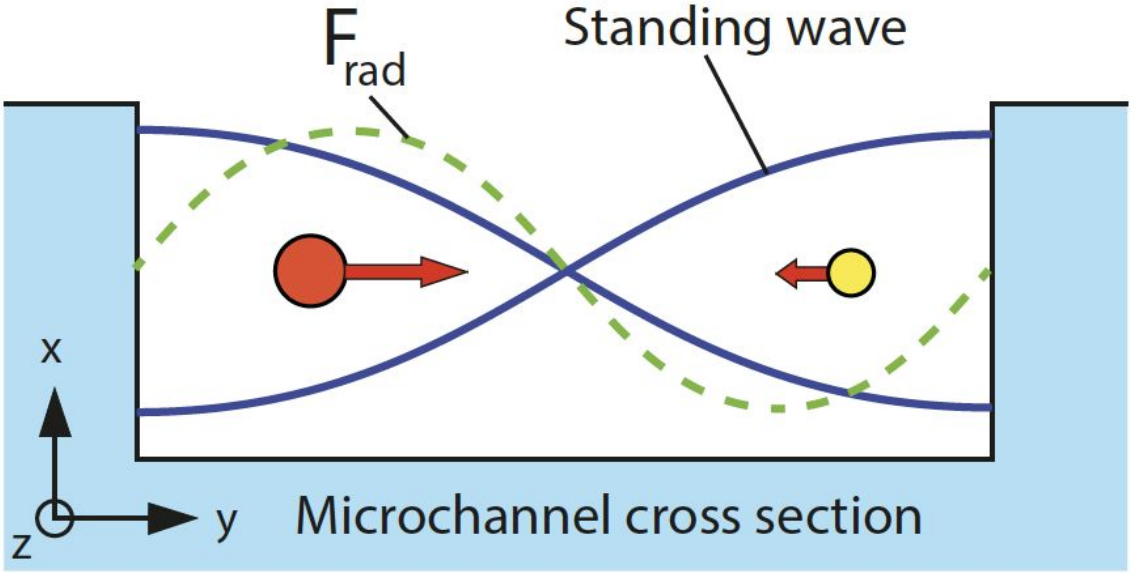

Figure S1. Schematic of the acoustophoresis principle, where cells with a higher acoustophoretic mobility migrates faster towards the standing wave pressure node in the center of the channel while following the flow in the z-direction to the outlets.

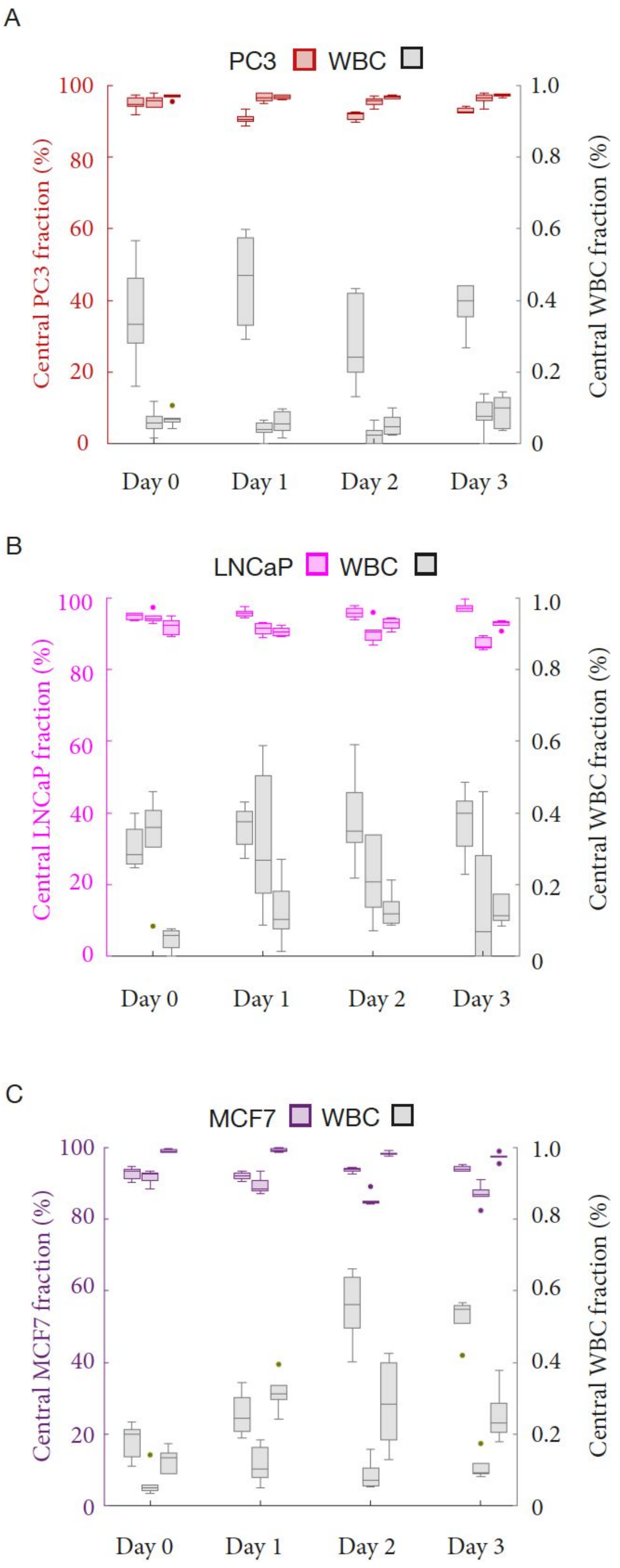
A
PC3
WBC
Central PC3 fraction (%)
Central WBC fraction (%)
100
80
60
40
20
0
1.0
0.8
0.6
0.4
0.2
0
Day 0
Day 1
Day 2
Day 3
B
LNCaP
WBC
Central LNCaP fraction (%)
Central WBC fraction (%)
C
MCF7
WBC
Central MCF7 fraction (%)
Central WBC fraction (%)

Figure S2. Acoustic separation performance for cancer cell line cells mixed with WBCs from healthy donors over time. (A)PC3, (B) LNCAP, and (C) MCF7. The horizontal line inside each box represents the median, top and bottom edges represent the upper and lower quartiles, whiskers indicate non-outlier minimum and maximum, and rings indicates values outside 1.5 times the interquartile range, (n=6).

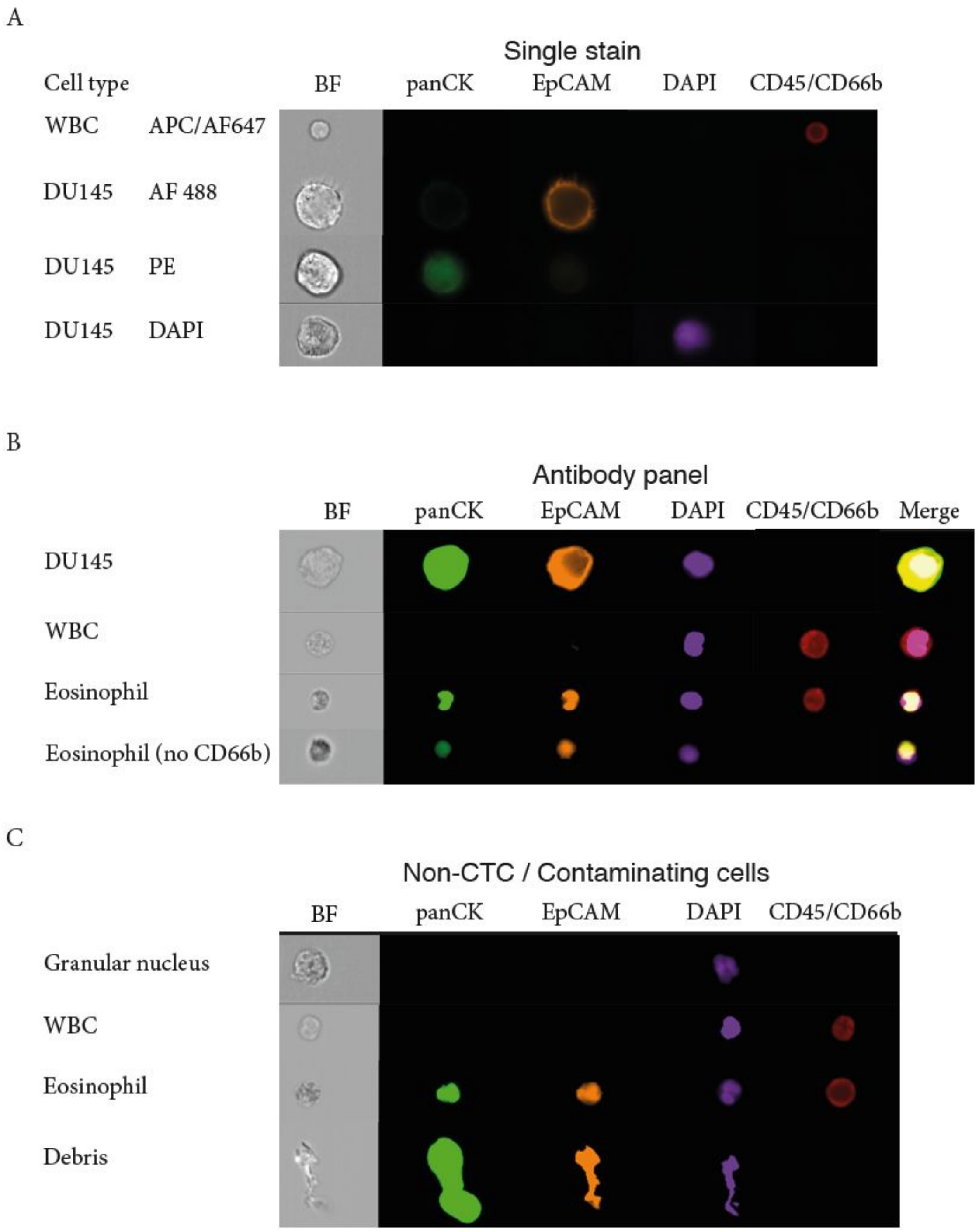

Figure S3. Control staining of DU145 cell line and WBCs from a healthy blood donor and contaminating non-CTC cells from mPCa patient samples. (A) Single antibody staining of control cells (B) Representative images using the full antibody panel. (C) Examples of representative contaminating cells found in the central fraction from patient #1, which were discarded as non-CTCs.

Supplementary note 1:

Theory behind acoustic cell separation:

Acoustophoresis utilizes the difference in acoustophoretic mobility between cell species, i.e., the acoustic radiation force, F_{rad} (eq. 1), acting on cells depending on the cells specific acoustophysical properties, i.e., size, density, and compressibility. Cells exposed to an acoustic standing wave, as illustrated in Fig 1B and C, will migrate to the center of the microchannel at a velocity, u_y (eq. 2), determined by its physical properties while following the flow along the channel. Cells that have a higher acoustophoretic mobility will migrate faster to the channel center and are collected at the central outlet, which is the case for the CTCs in this study. Slower migrating cells, predominantly WBCs will be discarded via the side outlet.

Equation (S1) shows the position (y_c) dependent force (F_{rad}) acting on a suspended cell of radius (a), density ρ_c, and compressibility κ_c in a plane standing wave of acoustic energy density E_{ac} with a pressure node located at $y = 0$ [1] .

$$F_{\text{rad}} = -4E_{\text{ac}}\, k_y\, \pi\, a^3 \Phi \sin(2k_y y_p) \qquad \text{(S1a)}$$

$$\Phi = \frac{1-\tilde{\kappa}}{3} + \frac{\tilde{\rho}-1}{2\tilde{\rho}+1} \qquad \text{(S1b)}$$

Here $k_y = 2\pi/\lambda$ is the wave vector and $\tilde{\kappa} = \kappa_c/\kappa_m$ and $\tilde{\rho} = \rho_c/\rho_m$ is the relative compressibility and density of the cell with respect to the suspending medium (indicated with superscript *m*).

The resulting velocity of the cell due to drag from the fluid is expressed in equation (S2).

$$u_y = F_{\text{rad}} / 6\pi\eta a \propto \Phi a^2 \qquad \text{(S2)}$$

The velocity of a cell is thus proportional to the square of the cell radius, which explains the strong dependency of the cell size, while differences in density and compressibility lead to a linear change.

Supplemental figure 1 schematically shows the cross section of a microchannel where a CTC and a WBC are exposed to the acoustic radiation force, resulting in a higher migration velocity (red arrow) to the channel center as compared to WBCs. Direction of buffer flow is in the z-direction.

Data analysis of discrimination between cancer cell line cells and white blood cells for increasing storage time in the acoustophoretic CTC chip:

To discriminate between cancer cell line cells and WBCs for increasing storage time, the number of cells of each type in the central (N_c) and side (N_s) outlet streams were counted after each run. The central fraction of a cell type (i) was calculated as the relative fraction ($f_{c,i}$) of that cell in the center *vs* all retrieved cells, *i.e.*, $f_{c,i} = N_{c,i} / (N_{c,i} + N_{s,i})$. The ideal separation outcome is thus f_c = 100% for cancer cells and f_c = 0% for WBCs.

To measure the deviation of f_c *vs* storage time in a donor specific manner, we computed the mean for the six technical repeats on day 0, $<f_c(0)>$, for each of the three donors, and subtracted that from $f_c(t)$ for all the different storage times. The drift vs storage time was then estimated as the slope, with 95% confidence interval (CI95), of $f_c(t) - <f_c(0)>$ after linear fitting.

Cell cluster movement during acoustophoresis:

The strong size dependency for cell movement in acoustophoresis makes this method particularly suitable for isolation of cell clusters. For a cluster of N cells, the acoustic migration velocity u_{ac} is expected to scale with $N^{2/3}$ such that even a cluster of just two cells will move towards the center at ~1.6 times the velocity of a single cell. Here we have assumed that (i) the force on the cluster increases with the total volume $V_{\mathrm{cluster}} = N \cdot V_{\mathrm{cell}}$, ($ii$) the shape of the cluster is approximated as a sphere of volume V_{cluster}, and (iii) the hydrodynamic drag force scales with the radius of a sphere.

References:

1. Bruus H. Acoustofluidics 7: The acoustic radiation force on small particles. *Lab on a chip* **2012**, *12*, 1014-1021.
